# Nutrition Literacy in Relation to Nutritional Status Among Community-Dwelling Older Adults in Istanbul, Türkiye: A Hierarchical Regression Analysis

**DOI:** 10.3390/nu18203428

**Published:** 2026-10-19

**Authors:** Öykü Altınok, Sezen Sevdin, Nadide Gizem Tarakçı Filiz, Bahar Yalçın Çeliktaş, Muazzez Garipağaoğlu

**Affiliations:** Department of Nutrition and Dietetics, Faculty of Health Sciences, Fenerbahçe University, 34758 Istanbul, Türkiye; sezen.sevdin@fbu.edu.tr (S.S.); nadide.tarakci@fbu.edu.tr (N.G.T.F.); baharyalcinn@gmail.com (B.Y.Ç.); muazzez.garipagaoglu@fbu.edu.tr (M.G.)

**Keywords:** anthropometry, nutrition literacy, nutritional status, older adults

## Abstract

**Background/Objectives**: Although nutritional status is central to healthy aging, little is known about how it relates to nutrition literacy in community-dwelling older adults. We examined this association and whether it persisted after sequential adjustment for sociodemographic, health-related, and anthropometric characteristics. **Methods**: This cross-sectional study included 306 community-dwelling adults aged ≥65 years in Istanbul, Türkiye. Nutritional status and nutrition literacy were assessed using the Mini Nutritional Assessment–Short Form (MNA-SF) and the Evaluation Instrument of Nutrition Literacy on Adults, respectively. Sociodemographic, perceived health- and nutrition-related, and anthropometric data were collected. Group comparisons, Pearson correlations, and a three-block hierarchical multiple regression analysis were performed. **Results**: Overall, 4.2% of participants were malnourished and 38.9% were at risk of malnutrition; 8.5% had inadequate and 66.7% had adequate nutrition literacy. Nutrition literacy was weakly and positively correlated with waist-to-hip ratio, mid-upper arm circumference, and calf circumference. Nutrition literacy was positively associated with MNA-SF scores across all hierarchical models, and the final model explained 28.8% of the variance. Sex, employment status, perceived diet quality, calf circumference, and waist circumference were also associated with MNA-SF scores in the final model. However, after additional adjustment for age group and marital status, the association between nutrition literacy and MNA-SF scores was no longer statistically significant. **Conclusions**: Nutrition literacy was modestly and positively associated with nutritional status after sequential adjustment, but not after additional adjustment for age group and marital status. Given the cross-sectional design, the direction of this association cannot be established, and no inferences regarding interventions can be drawn. Longitudinal studies are needed to clarify its temporal nature and underlying mechanisms.

## 1. Introduction

Population aging has increased the public health relevance of nutrition-related problems in later life [1]. This issue is particularly important for community-dwelling older adults who may remain outside routine nutritional screening systems despite being vulnerable to malnutrition and related adverse outcomes [2,3]. Globally, the number of individuals aged 60 years and older is projected to increase from 1.1 billion in 2023 to 1.4 billion by 2030, while in Türkiye, the proportion of older adults is projected to increase from 11.1% in 2025 to 13.5% by 2030 [1,4]. This demographic transition has important implications for healthy aging, as advancing age is often accompanied by chronic disease burden, functional decline, polypharmacy, changes in appetite and body composition, and increased vulnerability to nutritional problems [3,5].

Nutritional status is a critical component of health in later life and is closely associated with physical function, morbidity, mortality, quality of life, and healthcare utilization [2]. Although malnutrition is more commonly observed in institutional or hospital settings, community-dwelling older adults may also experience substantial nutritional risk, particularly when early indicators such as reduced food intake, unintentional weight loss, declining muscle mass, or social isolation are not systematically assessed [3,6,7]. Thus, the routine evaluation of nutritional status in community settings is an important component of preventive geriatric care and public health strategies targeting healthy aging [3,8].

Nutrition literacy has increasingly been recognized as a relevant and potentially modifiable factor in nutrition-related health outcomes [9,10,11]. It refers to the ability to access, understand, assess, and apply nutrition-related information in daily life, including the interpretation of food labels, dietary recommendations, and nutrition information sources [9,12]. Inadequate nutrition literacy has been associated with poorer dietary choices, lower diet quality, nutrient inadequacy, and less favorable nutrition-related behaviors, whereas higher nutrition literacy has been associated with healthier dietary patterns and better self-management of nutrition-related health conditions [13,14]. Nutrition literacy may be particularly relevant in older adults, for whom age-related changes in cognitive and functional capacity, chronic disease burden, and increasingly complex health-related decisions may affect the ability to translate nutrition information into daily dietary practices [9,10]. Accordingly, nutrition literacy may represent an important factor associated with nutritional vulnerability in community-dwelling older adults.

At the same time, nutritional status in older adults is multidimensional and cannot be considered independently of sociodemographic, health-related, and anthropometric characteristics [5]. Anthropometric indicators are simple, non-invasive, and practical measures that may support the identification of nutritional vulnerability in older adults. In addition to abdominal adiposity indicators, such as waist circumference (WC) and waist-to-hip ratio (WHR), muscle-related measures, such as mid-upper arm circumference (MUAC) and calf circumference (CC), may provide useful information on nutritional status and age-related changes in body composition [15,16]. Sociodemographic and health-related characteristics may likewise contribute to nutritional vulnerability and may partly account for observed differences in nutritional status among older adults [5]. Therefore, these characteristics should be considered when evaluating whether nutrition literacy is associated with nutritional status.

Although nutrition literacy and nutritional status have each been investigated in older populations, evidence remains limited regarding whether the association between nutrition literacy and nutritional status persists after adjustment for relevant characteristics that may influence nutritional vulnerability. In particular, the extent to which this association persists after accounting for sociodemographic and health-related characteristics and anthropometric indicators has not been sufficiently clarified [9,17]. In the present study, nutrition literacy was therefore considered the primary explanatory variable, while these characteristics were incorporated sequentially as adjustment domains within a hierarchical regression framework. The sequential organization of the models was intended to evaluate the robustness of the association between nutrition literacy and nutritional status across increasing levels of adjustment rather than to represent a causal or mediational pathway. Accordingly, this study aimed to examine the association between nutrition literacy and nutritional status among community-dwelling older adults and to determine whether this association remained after sequential adjustment for selected sociodemographic and health-related characteristics, followed by anthropometric characteristics. We hypothesized that nutrition literacy would be positively associated with MNA-SF scores and that this association would remain after adjustment for the selected sociodemographic, health-related, and anthropometric characteristics.

## 2. Materials and Methods

### 2.1. Study Design and Participants

This cross-sectional study enrolled 306 community-dwelling adults aged 65 years and older from Istanbul, Türkiye, between December 2025 and March 2026. Participants were recruited using a snowball sampling method. Initially, potentially eligible older adults known to the researchers were approached and invited to participate in the study. Subsequently, enrolled participants were asked to refer other older adults within their social networks who might meet the eligibility criteria. Referred individuals were contacted, assessed for eligibility, informed about the study, and invited to participate voluntarily. Ethical approval was obtained from the Fenerbahçe University Non-Interventional Clinical Research Ethics Committee (Approval No. 114.2025fbu), and the study was conducted in accordance with the Declaration of Helsinki. Moreover, written informed consent was obtained from all participants before data collection.

The inclusion criteria were being aged ≥65 years, living in the community, being able to communicate and complete the questionnaires, and agreeing to participate in the study. Individuals residing in long-term care institutions or hospitalized at the time of data collection were not eligible. The exclusion criteria were unable to communicate adequately or complete the questionnaires. Although no formal cognitive screening instrument was used, individuals who were unable to understand the questions or who provided inconsistent responses during data collection were excluded. Because the questionnaires were self-administered and anthropometric measurements required the comprehension of verbal instructions, individuals with visual or hearing impairments that precluded these procedures could not be included.

An a priori power analysis was performed using G*Power version 3.1.9.7 (F tests; linear multiple regression: fixed model, R^2^ increase) to determine the sample size required to detect the unique contribution of nutrition literacy in the final regression model. Assuming a small-to-medium effect size (f^2^ = 0.05), an α level of 0.05, a statistical power of 0.95, one tested predictor, and 22 total predictors, the minimum required sample size was 263 participants. The analytic sample of 304 participants included in the hierarchical regression analysis exceeded this requirement. A sensitivity analysis based on the same model specifications indicated that with 304 participants, the minimum detectable effect size was approximately f^2^ = 0.043.

### 2.2. Data Collection

Data were collected face-to-face by researchers. The participants’ sociodemographic characteristics and perceived health- and nutrition-related variables were assessed using structured questionnaire forms. Anthropometric measurements were obtained, and data were also collected using the Mini Nutritional Assessment–Short Form (MNA-SF) and Evaluation Instrument of Nutrition Literacy on Adults (EINLA).

#### 2.2.1. Sociodemographic Characteristics

The participants’ sociodemographic characteristics included age, sex, marital status, education level, employment status, and perceived financial situation. Information on chronic disease history, regular medication use, smoking status, and alcohol consumption was also collected.

#### 2.2.2. Perceived Health and Nutrition Measures

Perceived nutrition knowledge, perceived diet quality, and perceived health status were assessed using single-item, three-category questions. The response categories were poor, moderate, and good for perceived nutrition knowledge and unhealthy, moderate, and healthy for perceived diet quality and perceived health status [18,19].

#### 2.2.3. Anthropometric Measurements

The anthropometric measurements were obtained directly by the researchers according to standardized procedures [20,21]. Body weight, height, WC, hip circumference (HC), MUAC, and CC were measured. Body weight was measured using a portable digital scale, height with a stadiometer, and circumference measurements with a non-elastic tape. Circumference measurements were taken twice, and the average of the two measurements was used when the difference was less than 1 cm. If the difference exceeded 1 cm, the measurements were repeated, and the average of the closest two values was used. Body mass index was calculated as body weight in kilograms divided by height in meters squared (kg/m^2^), and WHR was calculated by dividing WC by HC.

For the regression analyses, anthropometric indicators were categorized using prespecified cut-off values to facilitate interpretation. This approach was preferred because these indicators are commonly interpreted in clinical and screening settings using established cut-off values, and because the WC and WHR cut-offs are sex-specific, allowing sex-appropriate risk classification within a single model. Mid-upper arm circumference (MUAC) was dichotomized at 22 cm (<22 cm vs. ≥22 cm), consistent with the anthropometric criteria of the full Mini Nutritional Assessment [22]. Calf circumference (CC) was categorized as low (<31 cm) or normal (≥31 cm), based on the 31-cm cut-off validated for the calf-circumference-based MNA-SF in community-dwelling older adults [23]. Waist circumference (WC) and waist-to-hip ratio (WHR) were categorized using established cut-off values for abdominal adiposity and cardiometabolic risk [20]. WC was classified as normal (<94 cm in men and <80 cm in women), increased risk (94–<102 cm in men and 80–<88 cm in women), or substantially increased risk (≥102 cm in men and ≥88 cm in women). WHR was classified as normal (<0.90 in men and <0.85 in women) or elevated (≥0.90 in men and ≥0.85 in women). These categories were used for analytical interpretation and were not considered stand-alone diagnostic criteria.

#### 2.2.4. Mini Nutritional Assessment–Short Form

Nutritional status was assessed using MNA-SF, a validated screening tool for older adults. The original MNA was developed by Guigoz et al. (1996) [24], whereas the MNA-SF was validated by Rubenstein et al. (2001) [25]. The MNA-SF comprises six items, with total scores ranging from 0 to 14. The study participants were classified as malnourished if they scored 0–7 points, at risk of malnutrition if they scored 8–11 points, and having a normal nutritional status if they scored 12–14 points [26]. The Turkish validity and reliability of the instrument in older adults have been established by Sarıkaya et al. (2015) [27]. For MNA-SF scoring, BMI was used rather than CC.

#### 2.2.5. Evaluation Instrument of Nutrition Literacy on Adults

Nutrition literacy was assessed using the Evaluation Instrument of Nutrition Literacy on Adults (EINLA), which was developed by Cesur et al. [28]. It comprises 35 items and covers five domains: general nutrition knowledge, reading comprehension, food groups, portion sizes, and numerical literacy and food label reading. The total scores range from 0 to 35, with higher scores indicating higher nutrition literacy. The study participants were classified as having inadequate nutrition literacy if they scored 0–11 points, borderline nutrition literacy if they scored 12–23 points, and adequate nutrition literacy if they scored 24–35 points [28]. In the present study, the scale had a Cronbach’s alpha coefficient of 0.895.

### 2.3. Statistical Analysis

Statistical analyses were performed using IBM SPSS Statistics (version 27.0). The continuous variables were expressed as mean ± standard deviation (SD), while the categorical variables were expressed as number and percentage. The distribution of the continuous variables was assessed using visual methods and skewness-kurtosis values. The homogeneity of variances was evaluated using Levene’s test. The independent sample *t*-tests and one-way analysis of variance were used to compare the EINLA and MNA-SF scores across sociodemographic characteristics and perceived health and nutrition measures. Where Levene’s test indicated unequal variances (*p* < 0.05), Welch’s correction was applied and the corrected statistics are reported. These univariate comparisons were considered exploratory. Therefore, no adjustment for multiple comparisons was applied, post hoc pairwise comparisons were not performed, and their results were interpreted with caution. The associations between nutrition literacy score and anthropometric measurements were assessed using Pearson correlation analysis.

To examine the association between nutrition literacy and nutritional status, a three-block hierarchical multiple regression analysis was performed, with the MNA-SF score used as the dependent variable. Nutrition literacy was entered in the first block as the primary explanatory variable. Selected sociodemographic and health-related characteristics were entered in the second block. Anthropometric indicators were entered in the third block. The primary hypothesis was tested using the coefficient for nutrition literacy in the final model (Model 3). The categorical variables were dummy coded before inclusion in the regression model, with reference categories specified in the regression table. Missing data in the hierarchical regression analysis were handled using listwise deletion. Two participants had missing data for perceived diet quality and were therefore excluded from the regression analysis, resulting in an analytic sample of 304 participants. For each model, unstandardized coefficients (B) with SE and 95% confidence intervals, standardized coefficients (β), R^2^, adjusted R^2^, change in explained variance (ΔR^2^), overall F statistics, F-change statistics, and *p* values were reported. The assumption of multicollinearity was assessed using the variance inflation factor (VIF), with a VIF of <10 accepted as the criterion. Collinearity diagnostics, including tolerance and VIF values for the predictors across hierarchical regression models, are presented in Supplementary Table S1. To evaluate whether the use of BMI in MNA-SF scoring influenced the findings, two sensitivity analyses were performed using the same three-block hierarchical regression model. First, the MNA-SF was rescored using CC instead of BMI (<31 cm = 0 points; ≥31 cm = 3 points), a validated alternative scoring option of the instrument [26]; in this analysis, CC was removed from the third block to avoid including the same measurement on both sides of the equation. Second, the anthropometric item was excluded from the MNA-SF, yielding a score ranging from 0 to 11. The results of the sensitivity analyses are presented in Supplementary Table S2. In an additional analysis, age group and marital status, both of which were associated with nutrition literacy and/or MNA-SF scores in the univariate analyses, were added to the second block, and the two sensitivity analyses were repeated with this extended model. In all analyses, statistical significance was set at a *p* value of <0.05.

## 3. Results

The study participants had a mean age of 72.19 ± 6.79 years, of whom 4.2% were identified as malnourished and 38.9% were at risk of malnutrition. In terms of nutrition literacy, 8.5% of the study participants were classified as having inadequate nutrition literacy, whereas 66.7% were classified as having adequate nutrition literacy.

Table 1 presents the characteristics of the study participants, together with the EINLA and MNA-SF scores according to these characteristics. EINLA scores differed significantly according to age, marital status, education level, employment status, perceived nutrition knowledge, perceived diet quality, perceived health status, and perceived financial situation. MNA-SF scores differed significantly according to age, marital status, education level, employment status, regular medication use, perceived nutrition knowledge, perceived diet quality, and perceived health status.

Table 2 shows the results of the correlation analysis between nutrition literacy and anthropometric measurements. No statistically significant correlation was observed between nutrition literacy and WC (r = −0.075, *p* = 0.190). However, weak positive correlations were observed between nutrition literacy and WHR (r = 0.140, *p* = 0.014), MUAC (r = 0.160, *p* = 0.005), and CC (r = 0.266, *p* < 0.001).

Table 3 presents the model statistics for the three-block hierarchical multiple regression analysis, while Table 4 presents the regression coefficients for each model. In Model 1, nutrition literacy was positively associated with MNA-SF scores and explained 5.6% of the variance in nutritional status [F(1, 302) = 18.081, *p* < 0.001; adjusted R^2^ = 0.053]. In Model 2, the addition of sociodemographic and health-related characteristics significantly increased the explained variance to 17.6% [F(17, 286) = 3.588, *p* < 0.001; adjusted R^2^ = 0.127, ΔR^2^ = 0.119, *p* = 0.001]. Although the magnitude of the association between nutrition literacy and MNA-SF scores decreased after adjustment, nutrition literacy remained positively and significantly associated with nutritional status. After anthropometric indicators were added in Model 3, the explained variance increased further to 28.8% [F(22, 281) = 5.162, *p* < 0.001; adjusted R^2^ = 0.232, ΔR^2^ = 0.112, *p* < 0.001]. Nutrition literacy remained positively associated with MNA-SF scores in the final model (β = 0.126). In addition, sex, employment status, perceived diet quality, CC, and WC were significantly associated with MNA-SF scores in the final model. Overall, the sequential models showed that the association between nutrition literacy and nutritional status persisted after adjustment for selected characteristics.

In the sensitivity analyses (Supplementary Table S2), BMI-based and CC-based MNA-SF scores were strongly correlated (r = 0.882, *n* = 306), and agreement in nutritional status classification was 86.6% (Cohen’s κ = 0.746). Nutrition literacy remained positively associated with nutritional status in the final model when the MNA-SF was scored using CC (β = 0.240, *p* < 0.001) and when the anthropometric item was excluded (β = 0.191, *p* = 0.003). In contrast, the contribution of the anthropometric block was smaller when the MNA-SF was scored using CC (ΔR^2^ = 0.050, *p* = 0.001) and was not statistically significant when the anthropometric item was excluded (ΔR^2^ = 0.026, *p* = 0.098). The substantially increased risk WC category remained positively associated with nutritional status in both sensitivity analyses. When age group and marital status were additionally included in the second block, the association between nutrition literacy and MNA-SF scores was attenuated and was no longer statistically significant in the final model (β = 0.075, 95% CI for B −0.015 to 0.062, *p* = 0.229). The attenuation was mainly attributable to age (β = 0.088, *p* = 0.155 when only age group was added), whereas the addition of marital status alone had a smaller effect (β = 0.101, *p* = 0.102). In this extended model, the association remained significant when the MNA-SF was scored using CC (β = 0.154, *p* = 0.013) or without the anthropometric item (β = 0.131, *p* = 0.042).

## 4. Discussion

This study examined whether nutrition literacy was associated with nutritional status among community-dwelling older adults, and importantly, whether this association persisted after sequential adjustment for selected sociodemographic, health-related, and anthropometric characteristics. The main finding was that nutrition literacy remained positively associated with MNA-SF scores across all hierarchical models, including the final model. However, this association was modest, and given the cross-sectional nature of the data, neither its direction nor causality can be inferred. The findings showed that 43.1% of the participants were either malnourished or at risk of malnutrition. Although the proportion of participants classified as malnourished in this community-dwelling sample was lower than that among those living in institutional care, the observed proportion indicates a considerable burden of nutritional risk in this population [7,29]. Indeed, in community-based samples, malnutrition may often be underdiagnosed due to inadequate screening, low awareness, and the risk being overlooked, particularly in overweight individuals [3,6].

This association persisted after sequential adjustment for the selected characteristics and in the sensitivity analyses addressing the use of BMI in MNA-SF scoring. One possible explanation is that limited nutrition literacy makes it harder to access, understand, and apply nutrition-related information in everyday decisions about food selection and dietary practices [9]. The reverse is also plausible: poorer health and nutritional status may limit older adults’ capacity to engage with nutrition-related information. The relationship may therefore be bidirectional, with either or both pathways contributing to the observed association. Previous studies have also reported associations between higher nutrition literacy and more favorable dietary behaviors and diet quality [13,17].

The magnitude of this association, however, was modest. Based on the unstandardized coefficient (B = 0.039), a 10-point higher EINLA score corresponded to an MNA-SF score approximately 0.4 points higher, which is a small difference relative to the 0–14 range of the instrument and the width of its classification categories. In the sensitivity analyses, the corresponding differences were approximately 0.5–0.8 points, still below one MNA-SF point. Statistical significance should therefore not be equated with clinical relevance, and the association is unlikely to be clinically meaningful at the individual level when nutrition literacy is considered alone. This is perhaps not surprising, given that nutritional status in older adults is shaped by many factors [5], each of which may contribute only modestly. Nutrition literacy may thus be more informative when considered together with other sociodemographic, health-related, and anthropometric characteristics than as a stand-alone indicator of nutritional vulnerability.

The association was no longer statistically significant after additional adjustment for age group and marital status, and age accounted for most of this attenuation. Advancing age has been associated with lower nutrition literacy [10,30], and older age is also strongly associated with limited health literacy, a closely related construct, particularly on tests of reading comprehension, reasoning, and numeracy [31], skills that are closely linked to cognitive function [32]. Older age is likewise associated with a higher risk of malnutrition [33], partly through chronic disease burden, functional decline, the anorexia of aging, and oral health problems such as tooth loss and chewing difficulties [3,5,34,35]. Part of the observed association may therefore reflect the fact that both variables are related to age. It is also possible that lower nutrition literacy is one of the routes through which age-related nutritional vulnerability develops, but such pathways cannot be reliably examined with cross-sectional data [36]. A weak association did persist in the extended model when the MNA-SF was scored without BMI, indicating that the findings also depend on how nutritional status is measured. Overall, these results call for a cautious interpretation of the association between nutrition literacy and nutritional status in older adults.

The univariate comparisons discussed below were exploratory and not adjusted for multiple testing, so they should be interpreted with caution, especially where they were not supported by the multivariable analysis. The rate of adequate nutrition literacy among older individuals in the present study was 66.7%, which was lower than the 83.7% reported in the adult population in Türkiye [37]. This pattern suggests that advancing age may be associated with lower nutrition literacy. Previous studies have reported that adults have higher levels of nutrition literacy compared with older adults and that nutrition literacy tends to decrease with advancing age within the older population [10,30]. Similarly, in the present study, both EINLA and MNA-SF scores differed across age groups. This finding is consistent with previous studies suggesting that the risk of malnutrition increases with advancing age [33]. These findings may be explained by numerous factors during the aging process, such as decreased appetite, chewing and swallowing problems, gastrointestinal changes, chronic disease burden, and social isolation, as well as changes in cognitive function, reduced access to information, and the increasing complexity of health-related decision-making processes [38,39].

In the present study, both nutritional status and nutrition literacy were found to be significantly higher among employed older individuals than those among non-employed individuals; employment was also positively associated with the MNA-SF scores in the multivariable model. This association may reflect differences in functional capacity, daily routine, social participation, socioeconomic resources, or access to information among older adults who remain employed [40,41]. In contrast, social isolation has been considered an independent risk factor for malnutrition in older individuals, and a lack of social interaction negatively affects nutritional and general health status by reducing food intake [40,42]. Indeed, it has been shown that employed individuals have higher levels of nutrition knowledge and literacy [43]. Similarly, the higher nutritional status and nutrition literacy observed among married participants in the univariate analyses may partly reflect social support, although marital status does not directly indicate living arrangements. Previous studies have consistently shown that married individuals have higher nutrition literacy, healthier dietary behaviors, and higher diet quality [10,43,44]. Living with a partner has been associated with the facilitation of processes such as food procurement, meal preparation, and sharing responsibilities related to nutrition [45]. In contrast, dietary diversity decreases, and more irregular dietary patterns are observed among older individuals living alone [46].

In the present study, nutrition literacy differed significantly across categories of perceived health status, perceived diet quality, and perceived nutrition knowledge. Previous studies have also suggested that inadequate nutrition knowledge in older individuals is associated with limited nutrition literacy [47]. It has been reported that nutrition literacy is associated with better health outcomes by increasing adherence to healthy dietary patterns and may positively affect individuals’ health perceptions [9,13]. Moreover, it has been reported that high nutrition literacy supports the self-management of chronic diseases and contributes to individuals’ more positive evaluations of their health status [48]. Within this framework, individuals’ subjective evaluations of health and nutrition may represent relevant correlates of nutrition literacy. Because these perceptions were measured at the same time as nutrition literacy, our data cannot show whether nutrition literacy shapes health perceptions or whether people who view their health more favorably are more inclined to engage with nutrition-related information. In the univariate analyses, perceived health status and perceived diet quality were associated with MNA-SF scores, and perceived diet quality remained associated in the final model. These results are consistent with previous studies suggesting that low perceived health status and inadequate diet quality are associated with the risk of malnutrition [7,49]. However, although nutritional status differed according to perceived nutrition knowledge in the present study, perceived nutrition knowledge was not associated with MNA-SF scores after multivariable adjustment. This suggests that nutrition knowledge alone may be insufficient to account for variation in nutritional status, which is shaped by the interaction of multiple factors such as functional capacity, chronic disease burden, psychological status, social support, and socioeconomic conditions [50]. Previous studies have also shown that the association between nutrition knowledge and diet quality may be limited, particularly in older individuals, and that it may not be a significant predictor of nutritional status on its own [14,51].

The anthropometric findings provide additional context for these results. Nutrition literacy was weakly and positively correlated with MUAC and CC, both practical indicators of nutritional and muscle status in older adults [52,53], and previous studies have reported similar associations between nutrition literacy, diet quality, lifestyle behaviors, and anthropometric characteristics [44,54]. The weak positive correlation with WHR requires more careful interpretation. A higher WHR reflects greater abdominal adiposity and is an indicator of increased cardiometabolic risk rather than of favorable nutritional status [20], so this finding should not be taken to mean that participants with higher nutrition literacy had a more favorable nutritional or health profile. The correlation also corresponded to only about 2% shared variance, and elevated WHR was not associated with MNA-SF scores in the final model. As abdominal adiposity is a multifactorial phenotype shaped by interactions between genetic predisposition and environmental and lifestyle factors [55], any relationship between nutrition literacy and WHR is likely to be indirect and influenced by factors beyond nutrition-related knowledge and skills.

The anthropometric results of the regression models should also be read in light of the structure of the MNA-SF. BMI contributes up to 3 of its 14 points, and WC, MUAC, and CC are related to overall body size to varying degrees, whereas WHR mainly reflects body fat distribution rather than overall body size [20]. Including these indicators as predictors may therefore introduce some conceptual overlap with the outcome. The sensitivity analyses are consistent with this: the anthropometric block significantly increased the explained variance in the main analysis, but its contribution was smaller when the MNA-SF was scored using CC and was no longer significant when the anthropometric item was excluded (Supplementary Table S2). The positive association between the increased-risk and substantially increased-risk WC categories and nutritional status, which may seem unexpected, can be partly explained in the same way. However, the substantially increased risk WC category remained positively associated with the MNA-SF scores in both sensitivity analyses, so this overlap does not fully account for the finding. It should not be interpreted as indicating that abdominal adiposity is nutritionally protective; rather, because the MNA-SF is mainly sensitive to low body weight, reduced food intake, and unintentional weight loss, malnutrition risk may be reflected differently in individuals with abdominal obesity [26], in whom it may also emerge in different patterns [56]. Low CC, in contrast, was negatively associated with nutritional status in the main analysis, consistent with its use as a simple screening indicator [57,58], but this association was not observed when the anthropometric item was excluded, suggesting that it also partly reflected shared body size. Overall, these findings underline the importance of interpreting anthropometric indicators together, rather than in isolation, when assessing nutritional status in older adults.

This study has several strengths. First, the hierarchical approach allowed the association between nutrition literacy and nutritional status to be examined across sequential levels of adjustment, showing that it remained statistically significant but modest after accounting for the selected sociodemographic, health-related, and anthropometric characteristics, while the additional analyses showed how sensitive it was to further adjustment for age. Second, anthropometric measurements were obtained directly by the researchers using standardized procedures, reducing potential measurement error associated with self-reported anthropometric data. Third, nutritional status and nutrition literacy were assessed using validated instruments, strengthening the methodological quality of the study. Finally, the focus on community-dwelling older adults provides relevant evidence for a population in which nutritional vulnerability may remain under-recognized in the absence of routine nutritional screening.

The main limitation of this study is its cross-sectional design, which means that neither the direction nor the causality of the association between nutrition literacy and nutritional status can be determined; the two may influence each other, and both may be shaped by unmeasured factors. Residual confounding by characteristics not included in the models also cannot be ruled out. Because BMI contributes to the MNA-SF score, overlap with body-size-related anthropometric indicators may have influenced the associations observed for these indicators, although the sensitivity analyses using CC-based and anthropometric item–excluded scores gave consistent results for nutrition literacy. Categorizing the anthropometric indicators, although based on established cut-offs, may also have resulted in some loss of information compared with using them as continuous variables.

Snowball sampling and recruitment from a single metropolitan area may have introduced selection bias and limit the generalizability of the findings to older adults in other geographic or socioeconomic settings. Because recruitment relied on participants’ social networks, socially isolated older adults and those with lower functional status or lower health or nutrition literacy may have been underrepresented. These subgroups may be at greater nutritional risk [42] and have lower nutrition literacy [59], so their underrepresentation may have narrowed the range of both variables, leading to an underestimation of the prevalence of malnutrition and affecting the size of the observed association. The findings may therefore mainly reflect relatively socially connected and functionally independent older adults. Generalizability may be further limited because cognitive function was not formally screened, and older adults whose visual or hearing impairments prevented participation could not be included. In addition, the EINLA was validated in adults aged 18–64 years who had completed at least primary school and has not been specifically validated in older adults [28]. Age-related cognitive, visual, and education-related factors may therefore have influenced performance on the instrument, although all participants had completed at least primary school and internal consistency in this sample was high.

Perceived nutrition knowledge, diet quality, health status, and financial situation were self-reported and may be affected by subjective reporting or social desirability bias. Finally, no objective or quantitative dietary intake data, such as food records, 24-h dietary recalls, food frequency questionnaires, or validated diet quality indices, were collected. Perceived diet quality was assessed with a single three-category question; single-item measures of this kind have shown evidence of construct validity as proxies for overall diet quality [18], but they cannot capture energy and nutrient intake or specific dietary patterns. We were therefore unable to examine whether the association between nutrition literacy and nutritional status operates through actual dietary behavior, a plausible pathway given previous reports linking nutrition literacy to diet quality [13]. Future studies combining nutrition literacy assessment with detailed dietary assessment, preferably using longitudinal designs, are needed to clarify this pathway.

## 5. Conclusions

In this cross-sectional study of community-dwelling older adults in Istanbul, Türkiye, nutrition literacy was positively but modestly associated with nutritional status after sequential adjustment for selected sociodemographic, health-related, and anthropometric characteristics. The association was no longer statistically significant after further adjustment for age group and marital status, suggesting that age-related differences may account for part of this relationship. Because the data are cross-sectional, neither the direction nor the causality of the association can be determined. On the basis of these findings, nutrition literacy may be more informative when considered alongside other characteristics than as a stand-alone indicator of nutritional vulnerability in this population. Longitudinal studies are needed to clarify the temporal relationship between nutrition literacy and nutritional status and the mechanisms that may underlie it.

## Figures and Tables

**Table 1:** Participant characteristics and EINLA and MNA-SF scores according to these characteristics.

			MNA-SF					
Variable		*n* (%)	Mean ± SD	Test Statistic	*p*	Mean ± SD	Test Statistic	*p*
Age	65–74 years	210 (68.6)	26.40 ± 6.03	F = 14.92 †	<0.001	12.10 ± 1.90	F = 12.59	<0.001
	75–84 years	76 (24.8)	22.64 ± 7.75			10.80 ± 2.31		
	≥85 years	20 (6.5)	19.10 ± 7.31			10.85 ± 3.01		
Sex	Female	177 (57.8)	25.29 ± 6.99	t = 0.87	0.385	11.62 ± 2.35	t = −0.82 †	0.415
	Male	129 (42.2)	24.59 ± 6.86			11.81 ± 1.89		
Marital status	Non-married	114 (37.3)	23.33 ± 7.86	t = −3.08 †	0.002	11.22 ± 2.40	t = −3.02	0.003
	Married	192 (62.7)	25.98 ± 6.14			11.98 ± 1.97		
Education level	Primary school	151 (49.3)	22.92 ± 7.13	F = 16.44 †	<0.001	11.41 ± 2.38	F = 3.86 †	0.023
	High school	72 (23.5)	26.19 ± 6.49			11.75 ± 2.01		
	Undergraduate or graduate	83 (27.2)	27.72 ± 5.72			12.18 ± 1.81		
Employment status	Non-employee	244 (79.7)	24.16 ± 7.01	t = −4.89 †	<0.001	11.51 ± 2.22	t = −3.03	0.003
	Employee	62 (20.3)	28.26 ± 5.57			12.44 ± 1.77		
Chronic disease history	No	65 (21.2)	24.98 ± 8.63	t = −0.01 †	0.992	12.08 ± 1.88	t = 1.59	0.114
	Yes	241 (78.8)	25.00 ± 6.42			11.60 ± 2.23		
Regular medication use	No	63 (20.6)	25.41 ± 8.60	t = 0.46 †	0.650	12.37 ± 1.75	t = 2.76	0.006
	Yes	243 (79.4)	24.88 ± 6.45			11.53 ± 2.23		
Smoking status	No	248 (81.0)	25.19 ± 6.98	t = 1.02	0.307	11.60 ± 2.21	t = −1.72	0.087
	Yes	58 (19.0)	24.16 ± 6.74			12.14 ± 1.93		
Alcohol consumption	No	242 (79.1)	24.62 ± 6.83	t = −1.86	0.064	11.64 ± 2.22	t = −0.86	0.392
	Yes	64 (20.9)	26.42 ± 7.19			11.91 ± 1.97		
Perceived nutrition knowledge	Poor	60 (19.6)	21.12 ± 6.94	F = 13.46	< 0.001	10.92 ± 2.64	F = 3.84 †	0.024
	Moderate	164 (53.6)	25.56 ± 6.02			11.80 ± 1.82		
	Good	82 (26.8)	26.72 ± 7.63			12.07 ± 2.31		
Perceived diet quality	Unhealthy	33 (10.9)	19.30 ± 7.78	F = 19.02 †	< 0.001	10.39 ± 2.57	F = 5.74 †	0.005
	Moderate	180 (59.2)	24.65 ± 6.77			11.72 ± 1.87		
	Healthy	91 (29.9)	27.71 ± 5.53			12.14 ± 2.40		
Perceived health status	Unhealthy	25 (8.2)	20.76 ± 7.10	F = 6.73	<0.001	10.36 ± 2.68	F = 10.94	<0.001
	Moderate	157 (51.3)	24.75 ± 6.40			11.45 ± 1.95		
	Healthy	124 (40.5)	26.15 ± 7.24			12.28 ± 2.16		
Perceived financial situation	Difficult	79 (25.8)	23.11 ± 7.10	F = 6.49	0.002	11.38 ± 2.33	F = 1.15 †	0.320
	Moderate	156 (51.0)	24.97 ± 6.79			11.85 ± 2.01		
	Comfortable	71 (23.2)	27.13 ± 6.54			11.77 ± 2.31		

EINLA, Evaluation Instrument of Nutrition Literacy on Adults; MNA-SF, Mini Nutritional Assessment–Short Form; SD, standard deviation; *t*, independent-samples *t*-test statistic; *F*, one-way ANOVA statistic. † Welch correction was applied. *p* values are unadjusted for multiple comparisons, and the comparisons should be considered exploratory.

**Table 2:** Correlations between nutrition literacy and anthropometric measurements.

Variable	Mean	SD	*r*	*p*
WC	93.202	13.826	−0.075	0.190
WHR	0.885	0.101	0.140	0.014
MUAC	28.478	4.725	0.160	0.005
CC	37.239	7.724	0.266	<0.001

CC, Calf circumference; MUAC, Mid-upper arm circumference; r, correlation coefficient; SD, standard deviation; WC, Waist circumference; WHR, Waist-to-hip ratio.

**Table 3:** Model statistics for the hierarchical multiple regression analysis.

Model	R^2^	Adj. R^2^	F	*p*	∆R^2^	F Change	df1	df2	*p*
1	0.056	0.053	18.081	<0.001	0.056	18.081	1	302	<0.001
2	0.176	0.127	3.588	<0.001	0.119	2.587	16	286	0.001
3	0.288	0.232	5.162	<0.001	0.112	8.843	5	281	<0.001

**Table 4:** Hierarchical regression models for the MNA-SF scores.

		Model 1				Model 2				Model 3			
Block	Predictor	B (SE)	β	95% CI for B	*p*	B (SE)	β	95% CI for B	*p*	B (SE)	β	95% CI for B	*p*
1	Nutrition literacy												
		0.074 (0.017)	0.238	(0.040, 0.109)	<0.001	0.044 (0.019)	0.142	(0.006, 0.082)	0.022	0.039 (0.019)	0.126	(0.002, 0.077)	0.041
2	Sociodemographic and health-related characteristics												
	Sex (ref: female)												
	Male					0.241 (0.245)	0.055	(−0.241, 0.724)	0.326	0.613 (0.248)	0.140	(0.125, 1.102)	0.014
	Educational status (ref: primary school)												
	High school					0.030 (0.325)	0.006	(−0.610, 0.671)	0.926	−0.121 (0.308)	−0.024	(−0.727, 0.484)	0.694
	Undergraduate or graduate degree					0.195 (0.319)	0.040	(−0.434, 0.824)	0.542	0.254 (0.306)	0.052	(−0.349, 0.856)	0.408
	Employment status (ref: non-employee)												
	Employee					0.370 (0.330)	0.069	(−0.279, 1.019)	0.263	0.708 (0.319)	0.131	(0.080, 1.336)	0.027
	Perceived financial situation (ref: difficult)												
	Moderate					0.409 (0.302)	0.094	(−0.186, 1.003)	0.177	0.297 (0.284)	0.068	(−0.263, 0.856)	0.298
	Comfortable					−0.356 (0.377)	−0.069	(−1.099, 0.387)	0.346	−0.357 (0.356)	−0.070	(−1.057, 0.343)	0.317
	Chronic disease history (ref: no)												
	Yes					0.314 (0.413)	0.059	(−0.499, 1.127)	0.448	0.016 (0.393)	0.003	(−0.756, 0.789)	0.967
	Regular medication use (ref: no)												
	Yes					−0.804 (0.414)	−0.150	(−1.618, 0.011)	0.053	−0.743 (0.392)	−0.139	(−1.515, 0.029)	0.059
	Perceived nutrition knowledge (ref: poor)												
	Moderate					0.439 (0.331)	0.101	(−0.213, 1.091)	0.186	0.266 (0.313)	0.061	(−0.350, 0.882)	0.396
	Good					0.398 (0.410)	0.081	(−0.409, 1.206)	0.333	0.091 (0.391)	0.019	(−0.678, 0.861)	0.815
	Perceived diet quality (ref: unhealthy)												
	Moderate					0.837 (0.476)	0.190	(−0.099, 1.773)	0.079	1.059 (0.450)	0.240	(0.174, 1.945)	0.019
	Healthy					0.776 (0.540)	0.164	(−0.286, 1.838)	0.152	1.041 (0.512)	0.220	(0.033, 2.050)	0.043
	Smoking status (ref: no)												
	Yes					0.738 (0.324)	0.134	(0.101, 1.375)	0.023	0.440 (0.307)	0.080	(−0.164, 1.045)	0.153
	Alcohol consumption (ref: no)												
	Yes					0.011 (0.324)	0.002	(−0.626, 0.648)	0.972	0.128 (0.307)	0.024	(−0.476, 0.731)	0.678
	Perceived health status (ref: unhealthy)												
	Moderate					0.564 (0.536)	0.130	(−0.492, 1.620)	0.294	0.150 (0.512)	0.035	(−0.858, 1.158)	0.769
	Healthy					1.262 (0.560)	0.286	(0.160, 2.363)	0.025	0.990 (0.533)	0.224	(−0.059, 2.040)	0.064
3	Anthropometric measurements												
	MUAC (ref: normal)												
	Low									−0.470 (0.279)	−0.100	(−1.020, 0.080)	0.094
	CC (ref: normal)												
	Low									−0.740 (0.347)	−0.121	(−1.422, −0.057)	0.034
	WC (ref: normal)												
	Increased risk									1.051 (0.318)	0.211	(0.424, 1.677)	0.001
	Substantially increased risk									1.530 (0.324)	0.347	(0.892, 2.168)	<0.001
	WHR (ref: normal)												
	Elevated									−0.383 (0.268)	−0.088	(−0.911, 0.145)	0.154

WC, Waist circumference; WHR, Waist-to-hip ratio; MUAC, Mid-upper arm circumference; CC, Calf circumference.

## Data Availability

The raw data supporting the conclusions of this article will be made available by the authors on request.

## Supplementary Materials

The following supporting information can be downloaded at: https://www.mdpi.com/article/10.3390/nu18203428/s1, Table S1: Collinearity diagnostics for predictors included in the hierarchical multiple regression models of MNA-SF scores; Table S2: Sensitivity analyses addressing the use of BMI in MNA-SF scoring and additional adjustment for age group and marital status.

## Abbreviations

The following abbreviations are used in this manuscript:

BMI	Body mass index
CC	Calf circumference
CI	Confidence interval
EINLA	Evaluation Instrument of Nutrition Literacy on Adults
HC	Hip circumference
MNA-SF	Mini Nutritional Assessment–Short Form
MUAC	Mid-upper arm circumference
SE	Standard error
VIF	Variance inflation factor
WC	Waist circumference
WHR	Waist-to-hip ratio
