# Supplementary material for: Nutrition Literacy in Relation to Nutritional Status Among Community-Dwelling Older Adults in Istanbul, Türkiye: A Hierarchical Regression Analysis
Source: Nutrients. 2026 Oct 19;18(20):3428. doi: 10.3390/nu18203428 (PMC13708202; doi:10.3390/nu18203428)
Supplement: Supplementary file 1 [file nutrients-18-03428-s001.zip › nutrients-4575285-supplementary.pdf]

**Table S1.** Collinearity diagnostics for predictors included in the hierarchical multiple regression models of MNA-SF scores.

| | | Model 1 | | Model 2 | | Model 3 | |
|---|---|---|---|---|---|---|---|
| **Block** | **Predictor** | **Tolerance** | **VIF** | **Tolerance** | **VIF** | **Tolerance** | **VIF** |
| **1** | **Nutrition literacy** | | | | | | |
| | | 1.000 | 1.000 | 0.757 | 1.320 | 0.680 | 1.471 |
| **2** | **Sociodemographic and health-related characteristics** | | | | | | |
| | Sex (ref: female) | | | | | | |
| | Male | | | 0.929 | 1.077 | 0.797 | 1.254 |
| | Educational status (ref: primary school) | | | | | | |
| | High school | | | 0.717 | 1.394 | 0.706 | 1.417 |
| | Undergraduate or graduate degree | | | 0.676 | 1.479 | 0.649 | 1.541 |
| | Employment status (ref: non-employee) | | | | | | |
| | Employee | | | 0.770 | 1.298 | 0.722 | 1.384 |
| | Perceived financial situation (ref: difficult) | | | | | | |
| | Moderate | | | 0.596 | 1.677 | 0.592 | 1.690 |
| | Comfortable | | | 0.533 | 1.875 | 0.527 | 1.896 |
| | Chronic disease history (ref: no) | | | | | | |
| | Yes | | | 0.474 | 2.109 | 0.461 | 2.168 |
| | Regular medication use (ref: no) | | | | | | |
| | Yes | | | 0.483 | 2.070 | 0.473 | 2.115 |
| | Perceived nutrition knowledge (ref: poor) | | | | | | |
| | Moderate | | | 0.499 | 2.006 | 0.490 | 2.040 |
| | Good | | | 0.410 | 2.439 | 0.397 | 2.520 |
| | Perceived diet quality (ref: unhealthy) | | | | | | |
| | Moderate | | | 0.249 | 4.018 | 0.245 | 4.089 |
| | Healthy | | | 0.223 | 4.494 | 0.217 | 4.606 |
| | Smoking status (ref: no) | | | | | | |
| | Yes | | | 0.840 | 1.190 | 0.820 | 1.219 |
| | Alcohol consumption (ref: no) | | | | | | |
| | Yes | | | 0.780 | 1.282 | 0.765 | 1.308 |
| | Perceived health status (ref: unhealthy) | | | | | | |

| | | Model 1 | | Model 2 | | Model 3 | |
|---|---|---|---|---|---|---|---|
| **Block** | **Predictor** | **Tolerance** | **VIF** | **Tolerance** | **VIF** | **Tolerance** | **VIF** |
| | Moderate | | | 0.189 | 5.291 | 0.182 | 5.483 |
| | Healthy | | | 0.180 | 5.562 | 0.174 | 5.745 |
| **3** | **Anthropometric measurements** | | | | | | |
| | MUAC (ref: normal) | | | | | | |
| | Low | | | | | 0.721 | 1.387 |
| | CC (ref: normal) | | | | | | |
| | Low | | | | | 0.788 | 1.269 |
| | WC (ref: normal) | | | | | | |
| | Increased risk | | | | | 0.619 | 1.615 |
| | Substantially increased risk | | | | | 0.470 | 2.127 |
| | WHR (ref: normal) | | | | | | |
| | Elevated | | | | | 0.672 | 1.487 |

WC, Waist circumference; WHR, Waist-to-hip ratio; MUAC, Mid-upper arm circumference; CC, Calf circumference; VIF, Variance inflation factor.

**Table S2.** Sensitivity analyses addressing the use of BMI in MNA-SF scoring and additional adjustment for age group and marital status

| | Main analysis: BMI-based MNA-SF (0–14) | Sensitivity analysis A: CC-based MNA-SF (0–14) [a] | Sensitivity analysis B: MNA-SF excluding the anthropometric item (0–11) |
|---|---|---|---|
| **A. Main model** | | | |
| Nutrition literacy, Model 1: B (SE); β; p | 0.074 (0.017); 0.238; <0.001 | 0.110 (0.018); 0.333; <0.001 | 0.076 (0.015); 0.273; <0.001 |
| Nutrition literacy, Model 2: B (SE); β; p | 0.044 (0.019); 0.142; 0.022 | 0.083 (0.020); 0.252; <0.001 | 0.051 (0.017); 0.181; 0.003 |
| Nutrition literacy, Model 3: B (SE); β; 95% CI; p | 0.039 (0.019); 0.126; (0.002, 0.077); 0.041 | 0.079 (0.020); 0.240; (0.039, 0.119); <0.001 | 0.053 (0.018); 0.191; (0.019, 0.088); 0.003 |
| Model 3: $R^2$ / adjusted $R^2$ | 0.288 / 0.232 | 0.256 / 0.201 | 0.236 / 0.176 |
| Anthropometric block: $\Delta R^2$ (F change; p) | 0.112 (8.843; <0.001) | 0.050 (4.776; 0.001) | 0.026 (1.882; 0.098) |
| Model 3, WC increased risk: B (SE); β; p | 1.051 (0.318); 0.211; 0.001 | 0.815 (0.338); 0.155; 0.017 | 0.340 (0.295); 0.076; 0.250 |
| Model 3, WC substantially increased risk: B (SE); β; p | 1.530 (0.324); 0.347; <0.001 | 0.753 (0.349); 0.162; 0.032 | 0.697 (0.300); 0.176; 0.021 |
| Model 3, low CC: B (SE); β; p | −0.740 (0.347); −0.121; 0.034 | — [a] | −0.208 (0.321); −0.038; 0.519 |
| Model 3, low MUAC: B (SE); β; p | −0.470 (0.279); −0.100; 0.094 | −0.832 (0.293); −0.167; 0.005 | −0.230 (0.259); −0.055; 0.375 |
| Model 3, elevated WHR: B (SE); β; p | −0.383 (0.268); −0.088; 0.154 | −0.314 (0.289); −0.068; 0.277 | −0.388 (0.249); −0.099; 0.120 |
| **B. Extended model additionally adjusted for age group and marital status [b]** | | | |
| Nutrition literacy, Model 3: B (SE); β; 95% CI; p | 0.023 (0.019); 0.075; (−0.015, 0.062); 0.229 | 0.051 (0.020); 0.154; (0.011, 0.091); 0.013 | 0.037 (0.018); 0.131; (0.001, 0.072); 0.042 |
| Model 3: $R^2$ / adjusted $R^2$ | 0.322 / 0.261 | 0.324 / 0.265 | 0.275 / 0.210 |
| Age 75–84 years (ref: 65–74 years): B (SE); β; p | −0.828 (0.282); −0.165; 0.004 | −1.111 (0.293); −0.210; <0.001 | −0.727 (0.261); −0.162; 0.006 |
| Age ≥85 years (ref: 65–74 years): B (SE); β; p | −0.635 (0.513); −0.071; 0.217 | −2.022 (0.523); −0.213; <0.001 | −0.945 (0.475); −0.118; 0.048 |
| Married (ref: non-married): B (SE); β; p | 0.429 (0.259); 0.095; 0.099 | 0.258 (0.272); 0.054; 0.343 | 0.400 (0.240); 0.099; 0.096 |

All models were adjusted for the same sociodemographic and health-related characteristics as in the main analysis (n = 304). a In sensitivity analysis A, the anthropometric item of the MNA-SF was scored using CC (<31 cm = 0 points; ≥31 cm = 3 points), and CC was removed from the third block to avoid including the same measurement on both sides of the equation (Model 3 in Panel A: $F(21,282) = 4.632$, $p < 0.001$). BMI-based and CC-based MNA-SF scores were strongly correlated ($r = 0.882$, n = 306), with 86.6% agreement in nutritional status classification (Cohen's $\kappa = 0.746$). b In Panel B, age group and marital status were additionally included in the second block of the model.

When only age group was added to the main model, the coefficient for nutrition literacy in Model 3 was B = 0.027 (SE = 0.019), β = 0.088, 95% CI −0.010 to 0.065, p = 0.155 ($R^2$ = 0.315); when only marital status was added, it was B = 0.032 (SE = 0.019), β = 0.101, 95% CI −0.006 to 0.070, p = 0.102 ($R^2$ = 0.300). BMI, body mass index; CC, calf circumference; CI, confidence interval; MUAC, mid-upper arm circumference; SE, standard error; WC, waist circumference; WHR, waist-to-hip ratio.
